# Surgical Free Gingival Root Coverage Graft Embedded in a Modified Subperiosteal Flap: A Novel Case Report

**DOI:** 10.1155/crid/3402443

**Published:** 2025-12-03

**Authors:** Bardia Vadiati Saberi, Dina Maleki, Soheil Taghavi Namin

**Affiliations:** ^1^Department of Periodontology, School of Dentistry, Guilan University of Medical Sciences, Rasht, Iran; ^2^Dental Sciences Research Center, School of Dentistry, Guilan University of Medical Sciences, Rasht, Iran; ^3^Residency of Periodontology, Department of Periodontology, School of Dentistry, Guilan University of Medical Sciences, Rasht, Iran

**Keywords:** case report, free gingival graft, gingival recession, root coverage

## Abstract

Gingival recession is a prevalent condition impacting extensive populations, resulting in functional and aesthetic issues. Root coverage periodontal plastic surgery techniques have been utilized for the management of gingival recession for an extended period. To overcome the limitations and disadvantages of the previous approaches, the current innovative technique was introduced requiring no specific instruments, providing direct vision of the surgical site, reducing the chances of damaging the adjacent anatomical structures, preserving the interdental papilla, and releasing the muscle tension of the flap base increasing the success of root coverage surgery.

## 1. Introduction

Gingival recession (GR), defined as the apical displacement of the gingival margin with associated clinical attachment loss, is a prevalent condition affecting diverse populations and leading to functional and aesthetic challenges [1–3]. According to the 2017 World Workshop classification, GR is influenced by multiple etiological factors, including inflammation, anatomical predispositions (e.g., thin gingival biotype, abnormal tooth positioning), direct trauma (e.g., malocclusion, aggressive toothbrushing), and iatrogenic factors (e.g., subgingival restorations, improper partial dentures) [4]. These factors contribute to complications such as dental hypersensitivity, root caries, and compromised aesthetics, necessitating effective periodontal plastic surgery interventions [5–7].

Root coverage procedures, such as the free gingival graft (FGG), subepithelial connective tissue graft (SCTG), coronally advanced flap (CAF), and guided tissue regeneration (GTR), have been widely employed to manage GR [8, 9]. Among these, CAF combined with SCTG is considered the gold standard due to its predictable outcomes, with reported root coverage rates of 6%–86% depending on defect characteristics and surgical technique [10, 11]. However, these techniques have notable limitations. FGG often results in poor aesthetic outcomes due to color mismatch and requires a sizable donor site, increasing postoperative morbidity [12–14]. SCTG, while effective, involves complex harvesting from the palate, leading to limited tissue availability and potential complications such as pain and bleeding [15–18]. The VISTA technique and tunnel approach, while minimally invasive, require specialized instruments, offer limited visibility, and risk damaging adjacent anatomical structures or the interdental papilla due to blind instrumentation or sulcular incisions [16, 17, 19, 20].

To address these challenges, we introduce a novel modified subperiosteal flap technique utilizing a de-epithelialized free gingival graft (DFGG). This approach offers several clinical benefits for practitioners: (1) simplified instrumentation, requiring only standard periodontal tools (e.g., 15c blade, periosteal elevator) rather than specialized equipment, making it accessible to a broader range of clinicians; (2) enhanced surgical visibility, achieved through a horizontal incision 0.5 mm apical to the mucogingival junction (MGJ), reducing the risk of damaging critical structures like the mental nerve; (3) preservation of interdental papilla, avoiding sulcular or papillary incisions to maintain aesthetic integrity; (4) tension-free flap design, achieved by combining full-thickness coronal and partial-thickness apical flap elevation to release muscle tension, promoting stable graft integration; and (5) reduced donor site morbidity, as the DFGG requires only superficial palatal tissue compared to deeper SCTG harvesting. These advantages make the technique particularly suitable for managing Miller Class I and II recessions in patients with thin gingival biotypes, as demonstrated in this case report.

## 2. Case Presentation

**Figure 1:**
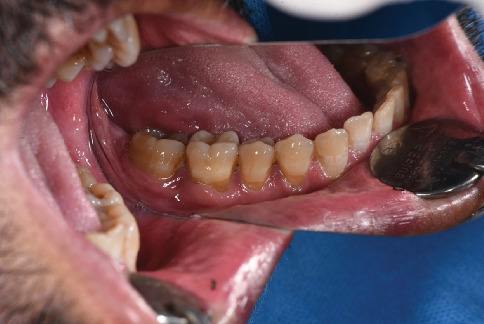
Teeth with gingival recession (Teeth 34, 35, and 36 according to the FDI numbering system).

A 54-year-old male patient was referred to the periodontal department of Guilan University of Medical Sciences. The patient's chief complaint was sensitivity to cold for the last 6 months and root visibility of mandibular left premolars and first molar (Teeth 34, 35, and 36 according to the FDI numbering system) (Figure 1). The patient has no history of systemic conditions, any drug or alcohol consumption, periodontal disease, or previous periodontal surgery. The patient had an aggressive tooth brushing habit, performing a horizontal tooth brushing technique using a hard toothbrush. Written informed consent, explicitly including permission to publish anonymized participant data and information in scientific publications, was obtained from the participant prior to their involvement. The study adhered to the Declaration of Helsinki.

After a primary clinical examination, the patient's oral hygiene was reported as good. The patient was diagnosed with generalized gingivitis. The involved teeth had no mobility, caries, or restoration. The exposed root surfaces were smooth and free of notable defects, indicating that restorative procedures, such as filling cervical abrasions, were not necessary prior to the root coverage surgery. The occlusion of the patient was not traumatic. No history of previous dental trauma was reported. According to the radiographical evaluation, no periodontal ligament (PDL) widening was observed around the involved teeth; the lamina dura was intact in the area; the trabecular bony pattern was normal; the bone architecture was scalloped in the area, and no sign of periapical changes was observed.

The gingival margin of the involved teeth was rounded. The keratinized width was 0 mm for Tooth 34, 2 mm for Tooth 35, and 2 mm for Tooth 36. The keratinized thickness was 0 mm for Tooth 34 and 1 mm for Teeth 35 and 36. The gingival phenotype was thin. The probing depth was 1–2 mm at the evaluated site. The GR of Teeth 34 and 35 was 3 and 2 mm, respectively. The GR of Tooth 36 was 3 mm at the mesial root and 1 mm at the distal root. No GR was beyond the MGJ. Interdental hard and soft tissue around the mentioned teeth was intact. According to Miller's classification of GR defects, the GR of Teeth 34, 35, and 36 was Class I. According to Jepsen's classification of GR defects, Teeth 34 and 35 were Recession Type 1 (RT1) and Tooth 36 was RT2. The overall prognosis of the patient was fair to good (Figure 1).

Phase 1 of periodontal treatment was performed. The patient was educated to perform the Bass tooth brushing technique with a soft toothbrush. The instructor emphasized the necessity of maintaining good oral hygiene. After 4 weeks of maintenance, the root coverage surgery was scheduled because the plaque index of the patient was lower than 10%.

Before the surgery, the treatment protocol was explained to the patient and written consent was obtained. Local anesthesia was performed by inferior alveolar nerve block and local infiltration techniques using two cartridges of lidocaine 4% with 1:80,000 epinephrine in the mandible (Darupakhsh, Iran). The local infiltration was conducted to prevent bleeding and provide better access. The donor site was the right side of the palate which was anesthetized by palatal infiltration technique using one cartridge of lidocaine 4% with 1:80,000 epinephrine.

**Figure 2:**
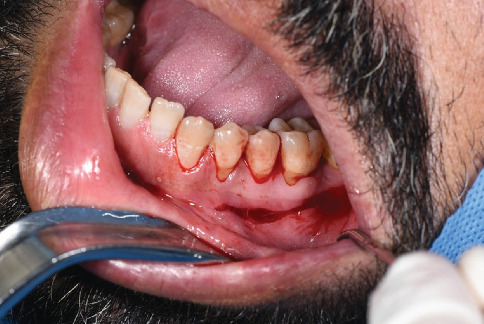
A horizontal incision was made 0.5 mm apical to the MGJ using a 15c blade from the distal root of Tooth 36 to the mesial root of Tooth 34.

**Figure 3:**
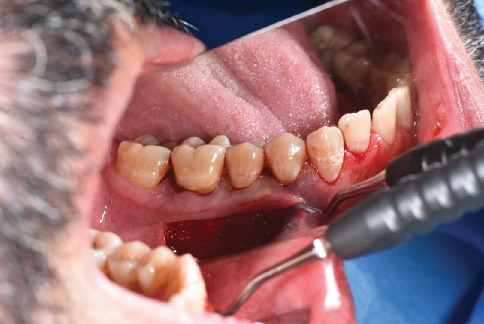
Coronally to the incision, the flap was elevated to full thickness. The elevation was continued to the base of the papilla.

**Figure 4:**
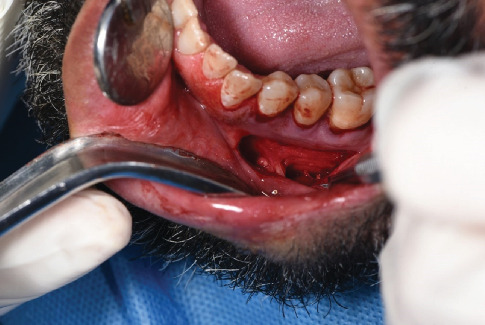
Apically to the incision, the flap was elevated in partial thickness to separate the muscle attachment. The mental nerve was preserved.

The denuded cervical surface of Teeth 34, 35, and 36 was thoroughly planed by area-specific curettes and was smoothened from residual necrotic cementum, toxins, and other irregularities without any use of chemical modifiers. A horizontal incision was made 0.5 mm apical to the MGJ using a 15c blade from the distal root of Tooth 46 to the mesial root of Tooth 34 (Figure 2). Coronally to the incision, the flap was elevated to full thickness. The elevation was continued to the base of the papilla (Figure 3). However, the interproximal papilla remained intact. Apically to the incision, the flap was elevated in partial thickness to separate the muscle attachment. The mental nerve was preserved (Figure 4).

**Figure 5:**
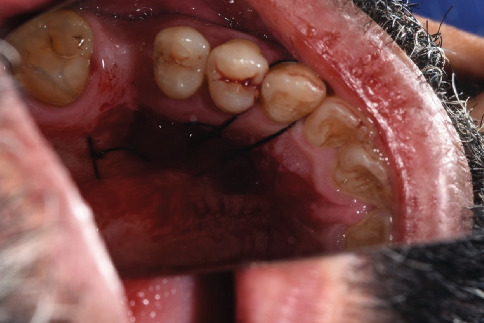
Palate donor site.

To harvest the graft from the palate, a 15c blade was used. An incision was made extending from Tooth 26 to Tooth 23 (according to the FDI numbering system), 2 mm apical to the gingival margin. Another incision was made perpendicular to the first incision extending for 6 mm. To elevate the graft, a small periosteal elevator was used. Following the harvest of the FGG from the palate, the donor site was closed using a crisscross suture technique with silk 3.0 sutures to approximate the wound edges and promote healing. One deep simple suture was placed at the distomedial angle of the donor site to secure the tissue and minimize bleeding. No additional covering materials, such as hemostatic agents or protective dressings, were applied, as the suturing was sufficient to stabilize the site. The patient was instructed to use chlorhexidine mouthwash (every 12 h for 2 weeks) to maintain oral hygiene and support healing at the donor site (Figure 5).

**Figure 6:**
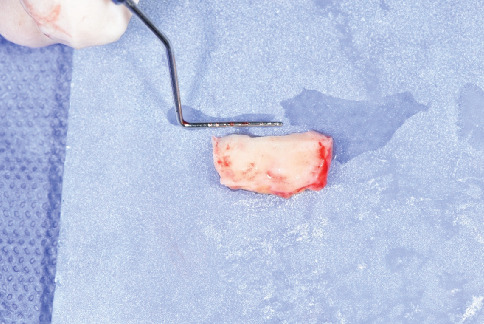
18 × 6 mm de-epithelialized free gingival graft.

**Figure 7:**
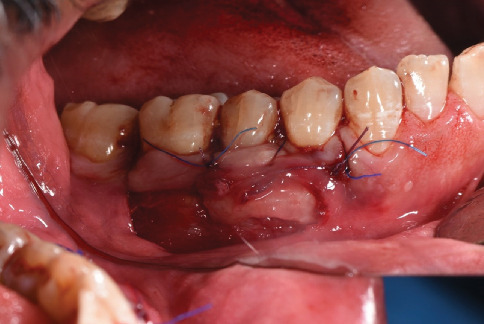
Simultaneously with the placement of the graft under the full-thickness flap in the mandibular surgical site, the flap was coronally displaced up to 2 mm over the cement-enamel junction and sutured using a simple suture technique by nylon suture 5.0.

**Figure 8:**
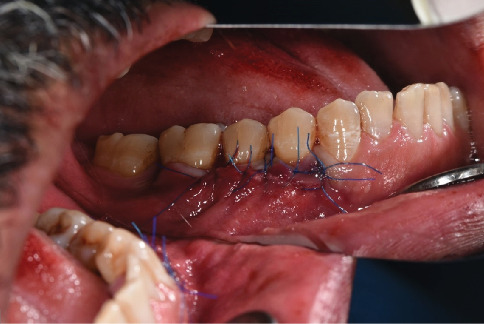
The apically partial-thickness flap was then sutured to the coronally full-thickness flap using a simple suture technique with nylon suture 5.0.

**Figure 9:**
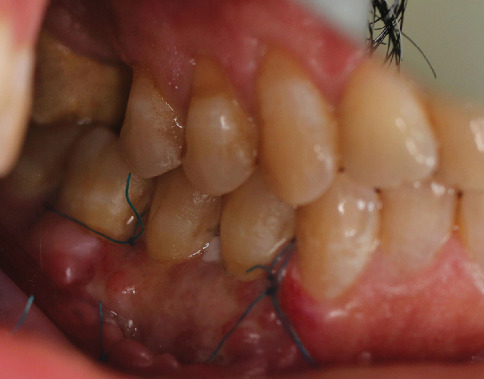
Immediately postsurgery ensuring complete coverage of the graft and tension-free adaptation. The flap was then stabilized in this coronally advanced position.

**Figure 10:**
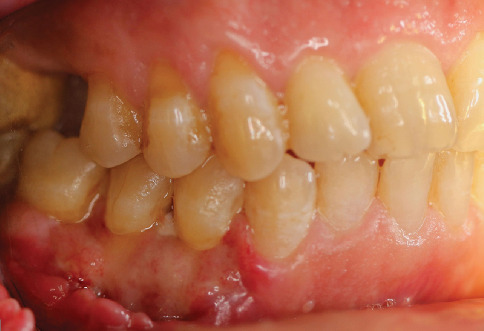
Two weeks postsurgery.

The obtained graft was 18 by 6 mm. A new 15c blade was used to de-epithelialize the graft to provide a DFGG (Figure 6). After placement of the 18 × 6 mm DFGG under the full-thickness flap in the mandibular surgical site, the flap was coronally repositioned to cover the graft and the exposed root surfaces of Teeth 34, 35, and 36. The coronal displacement was achieved by gently mobilizing the full-thickness flap up to 2 mm over the cement-enamel junction, ensuring complete coverage of the graft and tension-free adaptation. The flap was then stabilized in this coronally advanced position using a simple suture technique with nylon 5.0 sutures, with sutures placed to secure the flap to the surrounding tissue and maintain its position (Figure 7). Subsequently, the apically partial-thickness flap was sutured to the coronally full-thickness flap using the same simple suture technique with nylon 5.0 sutures to complete the closure and ensure stability of the surgical site (Figure 8). This suturing approach was aimed at stabilizing the flap and promoting integration; however, it likely contributed to localized tissue sloughing observed in the early postoperative period (Figures 9 and 10), which is consistent with the tissue maturation process induced by the DFGG's keratinizing effect on the overlying epithelium.

The patient was prescribed Novafen (capsule, every 8 h for 3 days) and chlorohexidine (mouthwash, every 12 h for 2 weeks). The patient was instructed to avoid tooth brushing the surgical site for the first 2 weeks postsurgery, brush the area using the Stillman tooth brushing technique with an ultrasoft toothbrush for the first 2 months postsurgery, and continue brushing his teeth using the Bass tooth brushing technique by a soft toothbrush after the first 2 months. The sutures were removed after 14 days.

**Figure 11:**
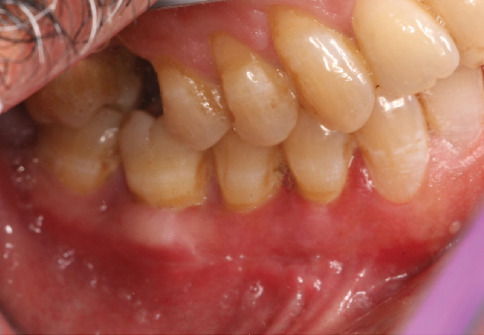
Four weeks postsurgery.

**Figure 12:**
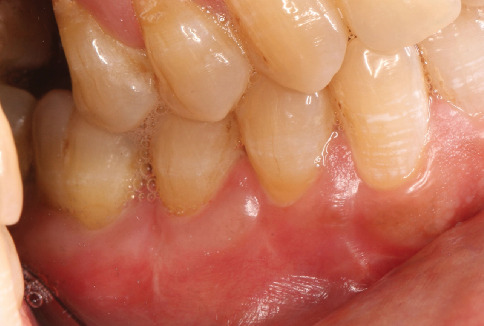
Eight weeks postsurgery.

The surgical site was followed at 2, 4, and 8 weeks (Figures 9, 10, 11, and 12). At 2 weeks postsurgery (Figure 10), localized sloughing of the superficial epithelium was observed, reflecting the expected remodeling process as the DFGG induced keratinization. By 4 weeks (Figure 11), the surgical site showed significant healing, with resolution of the sloughing and improved tissue integration. At 8 weeks (Figure 12), the sloughing had completely resolved, with the gingival margin appearing knife-edged, keratinized tissue widths of 2 mm (Tooth 34), 3 mm (Tooth 35), and 3 mm (Tooth 36); keratinized tissue thicknesses of 1 mm (Tooth 34) and 2 mm (Teeth 35 and 36); a thick gingival phenotype; probing depths of 1–2 mm; and GR reduced to 0 mm (Teeth 34 and 35) and 1 mm at the mesial root of Tooth 36, with an attachment gain of 2 mm (Tooth 34), 1 mm (Tooth 35), and 1 mm (Tooth 36).

## 3. Discussion

The modified subperiosteal flap technique with a DFGG presented in this case report introduces a novel approach to address GR, a prevalent condition that poses functional and aesthetic challenges for patients. This technique was developed to overcome limitations associated with established root coverage procedures, such as the vestibular incision subperiosteal tunnel approach (VISTA), the tunnel technique, and the CAF with connective tissue graft (CTG). By combining a full-thickness coronal flap with a partial-thickness apical flap and a strategically placed DFGG, this approach achieves tension-free flap adaptation, preserves the interdental papilla, and minimizes the risk of damaging adjacent anatomical structures, such as the mental nerve.

The VISTA technique is a minimally invasive method that involves a vertical vestibular incision and subperiosteal flap elevation, primarily for maxillary GRs [20–23] While effective for Miller Class I and II defects, VISTA requires specialized instruments and poses challenges due to limited visibility and blind instrumentation, which may increase the risk of gingival tearing or damage to adjacent structures [24–26]. In contrast, the current technique uses a horizontal incision 0.5 mm apical to the MGJ, providing direct visualization of the surgical site and eliminating the need for specialized instruments. This enhanced access reduces the risk of complications, such as mental nerve injury, and preserves the integrity of the interdental papilla, which is critical for maintaining aesthetic outcomes.

Similarly, the tunnel technique employs a partial-thickness envelope flap to facilitate CTG placement [27–29]. However, this approach often involves incisions through the gingival sulcus, which can compromise the interdental papilla and increase the risk of postoperative recession in these areas [28–30]. The current technique avoids sulcular incisions, preserving the papilla by elevating the flap from the MGJ, and releases muscle tension through a partial-thickness apical dissection, promoting stable graft integration [29, 30]. This tension-free flap design addresses a key factor in the failure of root coverage procedures, as flap tension has been associated with bone exposure, poor graft stabilization, and relapse [31, 32].

The CAF technique, often combined with CTG, is considered the gold standard for root coverage due to its predictable outcomes, with reported average root coverage ranging from 67% to 86% [33–36]. However, CAF requires incisions at the base of the papilla, which can lead to papillary recession and aesthetic compromise, particularly in patients with thin gingival biotypes [34, 36]. The modified subperiosteal flap technique mitigates this risk by avoiding papillary incisions and leveraging the DFGG to augment keratinized tissue thickness, as evidenced by the postoperative outcomes in this case (e.g., keratinized tissue width increased to 2–3 mm and a thick gingival phenotype at 8 weeks). The use of a DFGG, as opposed to a traditional CTG, also reduces donor site morbidity, as it eliminates the need for a deeper connective tissue harvest while still providing sufficient tissue volume for root coverage [37–39].

The clinical outcomes of this case, including complete root coverage for Teeth 34 and 35 and near-complete coverage for Tooth 36 (1-mm residual recession at the mesial root) at 8 weeks, compare favorably with the literature. For instance, studies on CAF with CTG report mean root coverage of 80%–90% for Miller Class I and II defects, with keratinized tissue gains similar to those observed here [40, 41]. The preservation of the interdental papilla and the absence of complications, such as graft mobility or postoperative infection, further highlight the potential advantages of this technique.

The biological rationale for this technique lies in its ability to optimize blood supply and graft integration. By elevating a full-thickness coronal flap, the periosteal blood supply is preserved, enhancing graft vascularization, while the partial-thickness apical flap releases muscle tension, reducing stress on the suture line [36, 39, 42]. The DFGG, derived from the palate, provides a robust source of keratinized tissue, which is critical for maintaining periodontal health in areas with minimal preoperative keratinized tissue (< 2 mm), as supported by Lang and Löe [43]. The absence of chemical modifiers during root planing, as used in this case, aligns with recommendations to minimize cytotoxicity and promote natural tissue healing [44–46].

Despite its advantages, the technique has potential challenges. The surgical procedure requires precise flap management to ensure tension-free closure, and the de-epithelialization of the FGG demands technical skill to avoid damaging the underlying connective tissue [33, 35]. Additionally, while the palatal donor site was managed with suturing alone in this case, alternative approaches (e.g., hemostatic agents or collagen matrices) may be considered to further reduce donor site morbidity [47]. The generalizability of this technique to more complex cases, such as Miller Class III or IV defects or patients with systemic conditions, remains to be explored, as this case involved a healthy patient with Miller Class I and RT 1–2 defects.

The modified subperiosteal flap technique offers a promising alternative for root coverage, particularly in cases with thin gingival biotypes and limited keratinized tissue. Its advantages include simplicity, accessibility, and reduced risk of complications compared to VISTA, tunnel, and CAF techniques. However, future studies are needed to validate its efficacy across a larger patient population and to assess long-term outcomes. Randomized controlled trials comparing this technique with CAF + CTG or other methods, with follow-up periods of at least 12 months, would provide stronger evidence of its clinical utility. Additionally, investigations into patient-reported outcomes, such as postoperative pain and aesthetic satisfaction, could further elucidate the technique's benefits.

## 4. Conclusion

The modified subperiosteal flap technique with a DFGG presented in this case report offers a promising approach for managing GR, addressing key limitations of existing methods such as the VISTA, tunnel technique, and CAF. Specifically, this technique minimizes the risk of damaging adjacent anatomical structures, such as the mental nerve, by utilizing a horizontal incision 0.5 mm apical to the MGJ, which provides direct visualization and controlled flap elevation, as demonstrated in the successful treatment of Miller Class I and RT 1–2 defects in Teeth 34, 35, and 36. This approach also eliminates the need for specialized instruments, preserves the interdental papilla, and achieves a tension-free flap through combined full- and partial-thickness flap elevation, contributing to stable graft integration and favorable clinical outcomes (e.g., complete root coverage for Teeth 34 and 35 and 1-mm residual recession at the mesial root of Tooth 36 at 8 weeks). While these results suggest that the technique is effective and safe, the findings are based on a single case with a 2-month follow-up, and further studies with longer-term follow-up and larger patient cohorts are needed to validate its efficacy and safety across diverse clinical scenarios.

## Data Availability

Date are available on request due to privacy/ethical restrictions.
